# Highly efficient and automated isolation technology for extracellular vesicles microRNA

**DOI:** 10.3389/fbioe.2022.948757

**Published:** 2022-08-10

**Authors:** Kaili Di, Boyue Fan, Xinrui Gu, Rongrong Huang, Adeel Khan, Chang Liu, Han Shen, Zhiyang Li

**Affiliations:** ^1^ Department of Laboratory Medicine, Affiliated Drum Tower Hospital, Medical School of Nanjing University, Nanjing, China; ^2^ Jiangsu Key Laboratory of Medical Science and Laboratory Medicine, School of Medicine, Jiangsu University, Zhenjiang, China; ^3^ State Key Laboratory of Bioelectronics, School of Biological Science and Medical Engineering, Southeast University, Nanjing, China

**Keywords:** extracellular vesicles, microRNA, Fe_3_O_4_@TiO_2_, isolation, lung cancer

## Abstract

MicroRNA (miRNA) in extracellular vesicles (EVs) has great potential to be a promising marker in liquid biopsy. However, the present EV isolation methods, such as ultracentrifugation, have complicated and long-time operation, which impedes research on EV miRNA. The downstream complex miRNA extraction process will also significantly increase the detection cycle and loss. We first established a simple automated technique to efficiently extract target miRNAs in EVs from plasma based on Fe_3_O_4_@TiO_2_ beads with high affinity and capture efficiency. We combined a heat-lysis method for quick and simple EV miRNA extraction and detection. The results indicated that our method has more RNA yield than TRIzol or a commercial kit and could complete EV enrichment and miRNA extraction in 30 min. Through the detection of miRNA-21, healthy people and lung cancer patients were distinguished, which verified the possibility of the application in clinical detection. The automated isolation technology for EV miRNA has good repeatability and high throughput, with great application potential in clinical diagnosis.

## Introduction

Extracellular vesicles (EVs) are vesicles released by most prokaryotic and eukaryotic cells containing proteins, lipids, and nucleic acids, which can be transported to distant cells (Yanez-Mo et al., 2015; Jan et al., 2019; Mathieu et al., 2019). EV-derived microRNAs (miRNAs) have been discovered to play an important role in gene expression regulation and disease progression (Yang et al., 2017). EV miRNAs have been extensively studied as a highly sensitive marker for different diseases, such as ovarian cancer (Maeda et al., 2020), lung cancer (Wu and Shen, 2020), and breast cancer (Volovat et al., 2020). Presently, several miRNA diagnostic kits have been developed and applied in clinical use. Given their unique high stability and specificity as biomarkers, EV miRNAs have great promise in early detection, prognosis, and monitoring of diseases (Preethi et al., 2022; Zhao et al., 2022).

EV isolation is a necessary and challenging step for research involving EV miRNAs. However, the lack of rapid and efficient techniques for EV isolation is a limitation that needs to be addressed urgently. Current isolation methods, including ultracentrifugation, size-exclusion chromatography, polymer precipitation, immunoaffinity etc., are time-limited, complex to operate, and require expensive equipment. After EV isolation, RNA extraction methods, such as TRIzol, will further reduce the extraction efficiency of EV miRNAs and prolong the detection period. In recent years, the development of various nanomaterials applied to detection technologies has brought convenience (Xiao et al., 2019; Yang et al., 2020). In particular, magnetic beads have shown great advantage as a fast and easy to operate detection method (Mou et al., 2015; Tang et al., 2018; Mou et al., 2019a; Mou et al., 2019b). Therefore, more efficient EV miRNA isolation methods should be developed and applied for EV analysis (Lu et al., 2021; Zhao et al., 2022). Titanium dioxide (TiO_2_) is a nanomaterial widely used in many fields (Xin et al., 2021; Chen et al., 2022a; Gao et al., 2022; Li et al., 2022) and can combine with phosphorylated amino acid residues, including serine, tyrosine, and threonine (Liu et al., 2015). The surface of the TiO_2_ beads is cationic and specifically binds to the phosphate groups on the phosphorylated modified peptide fragments. Recent researchers have suggested that TiO_2_ is able to combine with the phosphate groups on the lipid bilayer membrane of EVs (Gao et al., 2019; Chen et al., 2022b).

In our study, Fe_3_O_4_@TiO_2_ beads were used for EV rapid capture, and high capture efficiency of 80% was achieved. To better combine magnetic bead capture capacity and downstream biomarker detection for clinical application, we first designed an automated technique to efficiently extract target miRNAs in EVs from plasma. This unique approach involves three steps. First, EVs were specifically captured by Fe_3_O_4_@TiO_2_. Second, EVs were directly cleaved by heating and releasing the target miRNAs. Finally, quantitative reverse transcription PCR (RT-qPCR) was used to amplify heat-released miRNAs.

## Materials and methods

### Materials

Primers and phosphate-buffered saline (PBS) were purchased from Sangon Biotech Co., Ltd. (Shanghai, China). Fe_3_O_4_@TiO_2_ was obtained from Nanjing Xiuyuan Biotechnology Co., Ltd. (Jiangsu, China). Monoclonal anti-CD63 antibody, monoclonal calnexin antibody, and monoclonal TSG101 antibody were purchased from Abcam Co., Ltd. (Shanghai, China). A solution of 28 wt% ammonium hydroxide (NH_3_•H_2_O) and PKH26 Red Fluorescent Cell Linker Mini Kit were obtained from Sigma Co., Ltd. (Shanghai, China)., A 0.22-μm syringe-driven filter was purchased from Millipore (United States). Deionized water was processed by Ultrapure Millipore water (18.2 MΩ cm). High glucose Dulbecco’s modified Eagle’s medium (DMEM) and penicillin–streptomycin solution were purchased from Hyclone (UT, United States). Exosome-depleted fetal bovine serum (FBS) media supplement was purchased from System Biosciences, SBI (CA, United States).

All cell lines were purchased from the Shanghai Cell Bank, Chinese Academy of Sciences (Shanghai, China). Healthy human and lung cancer patient plasma samples were supplied by Nanjing University-affiliated Drum Tower Hospital. The samples were centrifuged at 1,500 g for 10 min at 4°C. The supernatant was collected and centrifuged at 2,000 g for 10 min at 4°C to remove platelets. The supernatant was centrifuged at 15,000 g for 30 min and stored at −80°C.

### Isolation of model extracellular vesicles

A549 non–small cell lung cancer cells (NSCLC) and BEAS-2B normal human bronchial epithelial cells (NHBES) were cultured in DMEM medium supplemented with 1% (v/v) penicillin–streptomycin and 10% (v/v) with standard fetal bovine serum (FBS), at 5% CO_2_ in culture bottles. When the cell density was up to 70%, we discarded the supernatant and washed the cells with PBS. Finally, cells were subcultured in DMEM containing 10% exosome-depleted FBS. The supernatant was collected after 24 h incubation.

Supernatants from the A549 and BEAS-2B cell cultures were collected and centrifuged at 300 g for 10 min to remove cell debris, followed by centrifugation at 2,000 g for 10 min. The supernatant was then transferred to ultracentrifuge tubes and centrifuged in an ultracentrifuge (Beckman Coulter, Brea, CA, United States) at 110,000 g at 4°C for 70 min twice. The pellet was resuspended in PBS and immediately stored at −80°C.

### Characterization of extracellular vesicles

The EVs captured on Fe_3_O_4_@TiO_2_ were eluted by 5% NH_3_•H_2_O for 10 min rotated on a rotator. After magnetic separation, the supernatant containing EVs was diluted with PBS and centrifuged at 2,000 g for 10 min in an Amicon Ultra100 kd ultrafiltration tube. EVs were recovered for subsequent analysis.

A total of 20 μl of the EV sample was incubated with 2% phosphotungstic acid on the copper mesh for 10 min at room temperature. After the copper mesh was dried, the EVs were photographed by transmission electron microscopy (TEM) (JEOL JEM-2100, Japan). For scanning electron microscopy (SEM) (Hitachi, Japan), 10 μl of EV sample–enriched Fe_3_O_4_@TiO_2_ was added to the clean tin foil paper. After drying at room temperature, the tin foil paper was glued to the conductive tape and photographed.

The particle size and concentration of the EVs were analyzed by using a ZetaView nanoparticles tracking analyzer (Particle Metrix, Germany). The EV sample was diluted with 1 × PBS to an appropriate concentration (10^7^ ∼ 10^9^/ml). Data were recorded and analyzed at 11 positions.

### Laser scanning confocal microscope

A laser scanning confocal microscope is used to observe the combination of stained EVs and magnetic beads. The model EVs from the A549 cell were incubated with 0.5 mg Fe_3_O_4_@TiO_2_ beads for 10 min. After isolation, 20 μl of PE-labeled anti-CD63 antibodies was incubated with beads for 30 min in the dark. The beads were cleaned with 200 μl of PBS three times to remove uncombined antibodies. Fluorescence was observed and photographed by using a confocal laser microscope (Leica, Germany).

### Extracellular vesicle microRNA extraction and detection

**FIGURE 1:**
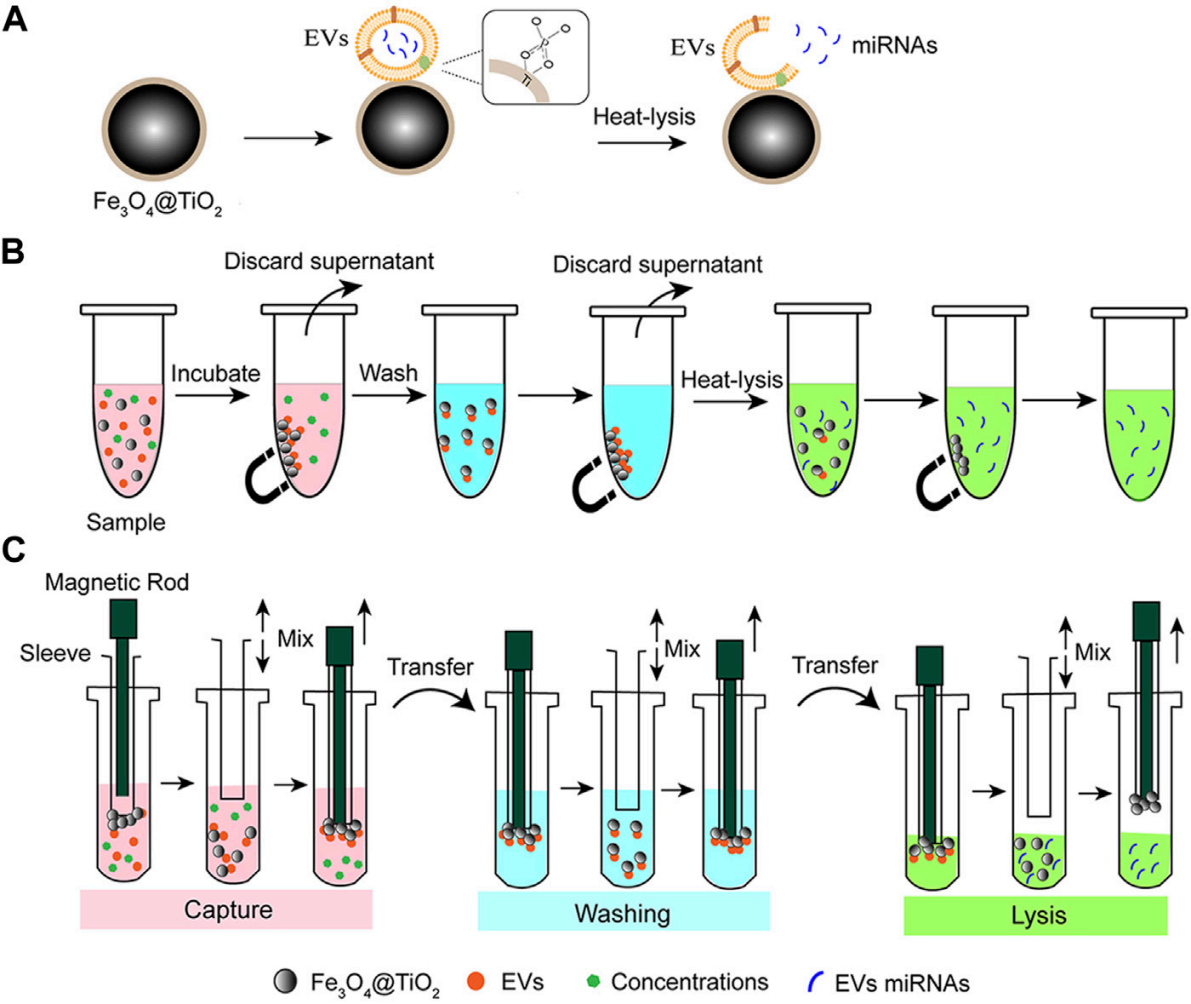
Schematic of EV miRNA isolation based on Fe_3_O_4_@TiO_2_. **(A)** Principle for EV isolation based on Fe_3_O_4_@TiO_2_. After capture, miRNAs were released from the EVs by the heat-lysis method. **(B)** Process of manual EV miRNA isolation. The sample was incubated with Fe_3_O_4_@TiO_2,_ and the beads were separated with magnets. Then, the supernatant was discarded after cleaning. Elution buffer was added to the beads to extract miRNAs. **(C)** Process of automated EV miRNA isolation. Binding buffer (pink), cleaning buffer (blue), and elution buffer (green).

A schematic of the automatic isolation and detection of EVs based on Fe_3_O_4_@TiO_2_ is shown in Figure 1C. First, the magnetic rod was used to transfer Fe_3_O_4_@TiO_2_ to the sample. The magnetic rods were added on the cover immersed in the tube, followed by a frame shake up and down of the covers for better incubation and mixing. Upon completion of incubation, the magnetic rods dropped down into the cover. Once they slide in, the magnetic beads bind the cover again. The magnetic rods transfer the attached beads and the bound EVs into another tube containing the washing solution. Finally, the beads and the captured EVs were transferred into the elution solution for heat-lysis. The beads were removed, and the miRNAs from the EVs were left in the solution. To evaluate the recovery rate for the automatic instrument, we calculated the difference in the weight of the beads before and after the experiment. The miRNA-21 expression level was detected by RT-qPCR to evaluate the extraction effect. The primer sequences of RT-qPCR are shown in Supplementary Table S1.

A total of 500 μl of the A549 cell supernatant was incubated with Fe_3_O_4_@TiO_2_. Different methods were used to compare the extraction effect of miRNA, including the heat-lysis method, TRIzol, and the miRNeasy Serum/Plasma Advanced Kit (Qiagen, Germany). In the heat-lysis method, 50 μl of RNase-free water was added to the magnetic beads after EV enrichment and heated in a metal bath at 95°C for 5 min. After magnetic separation, the supernatant was transferred for miRNA detection. For the TRIzol method, 600 μl of TRIzol lysis solution was added to the magnetic beads and then mixed with chloroform. After centrifugation, the upper aqueous phase was transferred and mixed with 500 μl isopropanol.Eighty percent ethanol was used to wash the RNA, followed by pouring out the supernatant carefully and drying precipitation. To dissolve the RNA, 50 μl of RNase-free water was added. For the commercial kit method, the RNA extraction followed steps miRNeasy Serum/Plasma as per the advanced kit instruction. Finally, 50 μl of RNase-free water was added, followed by centrifugation at full speed for 1 min to elute miRNA.

### Extracellular vesicle miRNA-21 analysis

First, the expression differences of miRNA-21 in EVs from A549 cells and BEAS-2B cells were compared. A total of 500 μl of the supernatant from each cell was incubated with 1 mg Fe_3_O_4_@TiO_2_ for 10 min. After washing, 50 μl RNase-free water was added to the magnetic beads, and miRNA was extracted using the heat-lysis method. Quantitative detection of miRNA-21 was performed by RT-qPCR. Each experiment was repeated three times.

An amount of 1 mg Fe_3_O_4_@TiO_2_ was used to enrich the EVs from the plasma of lung cancer patients and healthy persons. Next, 50 μl of RNase-free water was added to the magnetic beads, and miRNA was extracted by the heat-lysis method. Quantitative detection of miRNA-21 was performed by RT-qPCR, and the results were normalized with U6 as an internal reference.

## Results

### Extracellular vesicle enrichment based on Fe_3_O_4_@TiO_2_

The principle for EV isolation based on Fe_3_O_4_@TiO_2_ is shown in Figure 1A. Fe_3_O_4_@TiO_2_ beads were spherical and monodispersed under electron microscopy (Supplementary Figure S1). The diameter of Fe_3_O_4_@TiO_2_ was observed to be approximately 300–500 nm. The elemental composition of magnetic beads is shown in the energy-dispersive X-ray (EDX) spectrum (Supplementary Figure S2). The model EV samples were prepared by ultracentrifugation from the A549 cell supernatant. As shown in Figure 1B, 1 mg Fe_3_O_4_@TiO_2_ was added to 100 μl model EVs and incubated for 10 min on a rotator at room temperature. After incubation, the beads were separated and washed with PBS three times and finally resuspended in 100 μl PBS buffer.

**FIGURE 2:**
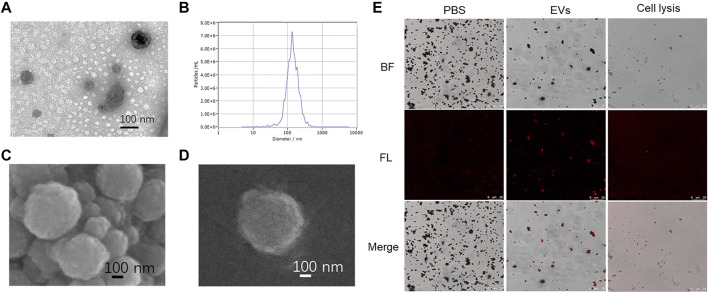
Characterization of EVs captured by Fe_3_O_4_@TiO_2_. **(A)** TEM image of model EVs. **(B)** Particle size analysis of EVs. **(C)** SEM image of Fe_3_O_4_@TiO_2_ after capture. **(D)** SEM image of single Fe_3_O_4_@TiO_2_ after capture. **(E)** Confocal laser scanning microscope image of Fe_3_O_4_@TiO_2_ captured with PBS, EVs, and cell lysis. EVs were stained with PE-labeled anti-CD63 antibody (red).

TEM images showed that the EVs maintained a round-cup morphology typical structure (Figure 2A), and the EVs were 50–150 nm in diameter as detected by NTA (Figure 2B). The Fe_3_O_4_@TiO_2_ beads captured with EVs were characterized by SEM (Figures 2C,D). The surface of the Fe_3_O_4_@TiO_2_ beads became rough because they combined with EVs. After Fe_3_O_4_@TiO_2_ was captured with PBS, EVs, and cell lysis, the beads were stained with the PE-labeled anti-CD63 antibody. The confocal laser scanning microscope (CLSM) pictures showed that red fluorescence was only observed when Fe_3_O_4_@TiO_2_ was incubated with EVs, indicating that Fe_3_O_4_@TiO_2_ had high affinity and specificity for EV capture (Figure 2E).

### Optimization of capture conditions

To obtain high capture efficiency, we optimized capture conditions according to a previous research method (Pang et al., 2020). PKH26-stained EVs were incubated with Fe_3_O_4_@TiO_2_ under different capture conditions. The fluorescence intensity for the supernatant after magnetic separation was detected. We optimized the amount of Fe_3_O_4_@TiO_2_, incubation time, and pH value for the binding buffer. After incubation of EV samples and Fe_3_O_4_@TiO_2_, the fluorescence intensity of the supernatant was detected after magnetic separation by using SpectraMax M5 Microplate Readers (Molecular Devices, United States).

**FIGURE 3:**
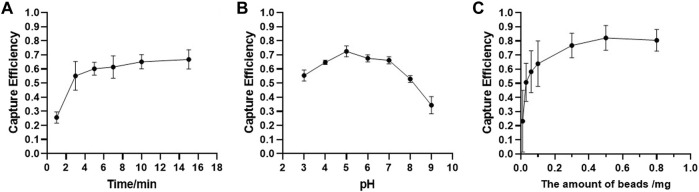
Optimization results for Fe_3_O_4_@TiO_2_ capturing conditions. **(A)** Optimization results for incubation time. **(B)** Optimization results for binding buffer pH value. **(C)** Optimization results for bead amount.

The capture efficiency was calculated according to the ratio of the fluorescence value in the supernatant and fluorescence value in the initial EV sample. As shown in Figure 3A, the capture efficiency increased with increased incubation time, and we obtained the best capture efficiency in 10 min. Buffer pH had a significant effect on the capture process. We obtained the best capture efficiency at pH value 5 (Figure 3B), which may be due to the positive charge of Fe_3_O_4_@TiO_2_, making it easier to combine with the EVs. The capture efficiency increased as the Fe_3_O_4_@TiO_2_ amount was increased from 0.01 to 0.8 mg. For further downstream experiments, 0.5 mg was the best fit (Figure 3C). The maximum capture efficiency reached 80%. In addition, according to SDS-PAGE results, Fe_3_O_4_@TiO_2_ beads can remove most impurity proteins in samples (Supplementary Figure S3). It indicated that Fe_3_O_4_@TiO_2_ beads have high affinity and specificity for EVs.

### Automatic extracellular vesicle microRNA extraction

**FIGURE 4:**
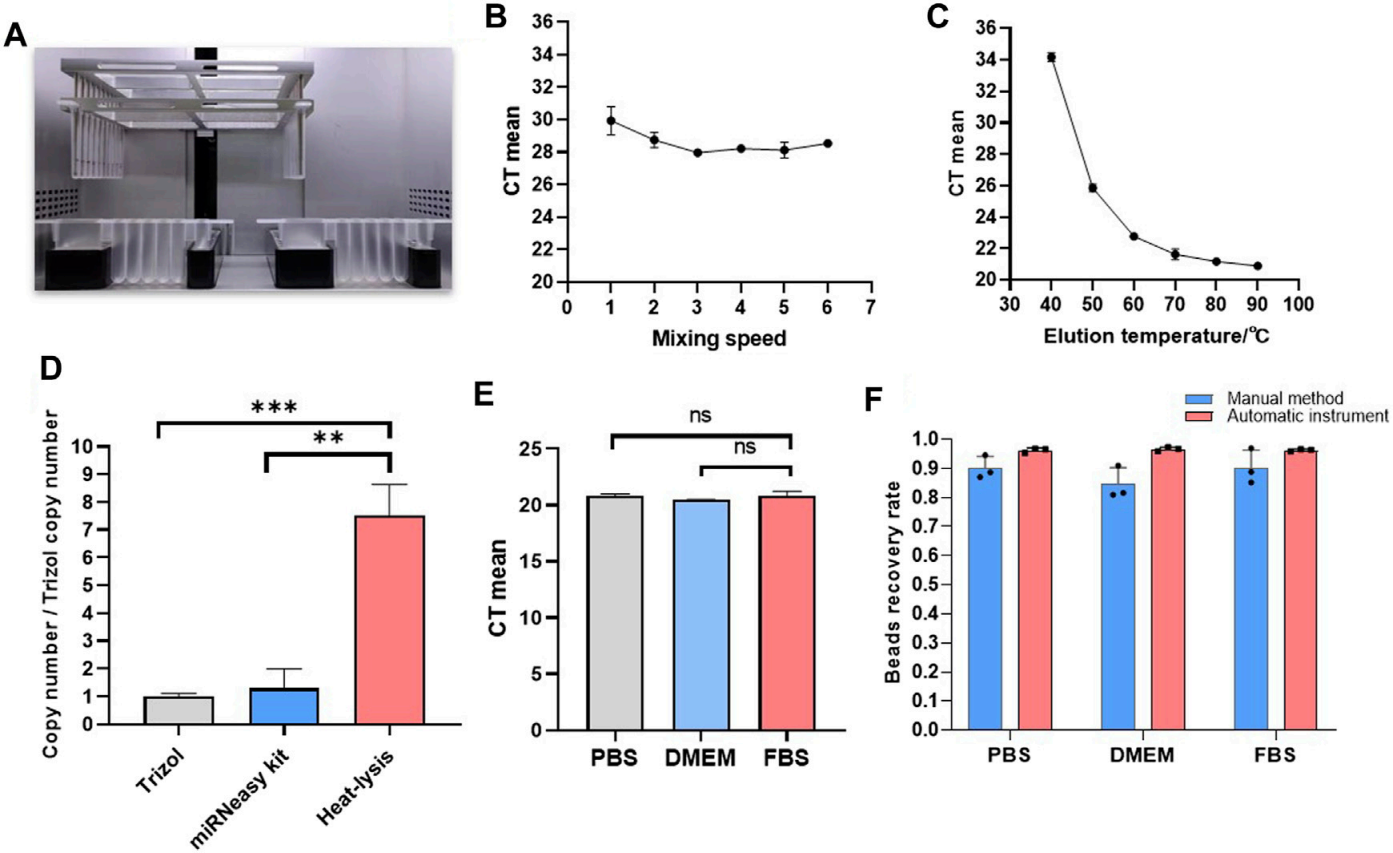
Automated EV isolation and miRNA extraction. **(A)** Internal structure of the automated instrument. **(B)** Optimization results for elution temperature. **(C)** Optimization results for mixing speed. **(D)** Copy number of miRNAs from different EV miRNA extraction methods. **(E)** CT values from EV miRNA extraction in different matrixes. **(F)** Recovery rate for magnetic beads by the manual method and automatic method (*p < 0.05, **p < 0.01, and ***p < 0.001, ns, non-significant).

A strategy for automated extraction of EV miRNA was proposed by combining the automatic instrument for the EV extraction method based on Fe_3_O_4_@TiO_2_ (Figure 1C). Figure 4A is the internal structure of the automatic instrument. In short, Fe_3_O_4_@TiO_2_ beads were adsorbed onto magnetic rods in the machine that can help in the separation and cleaning of the beads. First, the magnetic rod was used to transfer the magnetic beads to the sample. The magnetic rods were added on the cover immersed in the tube, followed by a frame shake up and down of the covers for better incubation and mixing. Upon completion of incubation, the magnetic rods dropped down into the cover. Once they slide in, the magnetic beads bind the cover again. The magnetic rods transfer the attached beads and the bound EVs into another tube containing the washing solution, and the frame moves up and down for better cleaning. Finally, the beads and the captured EVs were transferred by the magnetic rod for heat-lysis. With the help of the magnetic rod, the beads were removed and the nucleic acid from the EVs was left in the solution.

Before RT-qPCR detection and analysis, the standard curve of miRNA-21 was performed to verify the reliability of the primers (Supplementary Figure S4). Good linearity and a single product band indicated that it could be used for the detection of miRNA-21. To find the optimal condition in the automated equipment, the miRNA extraction effects were compared to different mixing speed level and temperatures. The grade 3 mixing speed was selected because the CT mean value for miRNA-21 was minimum (Figure 4B). The CT value decreased gradually when the temperature was increased (Figure 4C). So, we selected 90°C as the best lysis temperature. For verifying the advantages of this method in extraction, we compared the extraction effect of the heat-lysis method with TRIzol and the miRNeasy Serum/Plasma Advanced Kit. As shown in Figure 4D, the copy number of the heat-lysis method was about 7.5 times that of the TRIzol method and 5.8 times that of the kit method. Therefore, the heat-lysis method had better performance in EV miRNA extraction.

**FIGURE 5:**
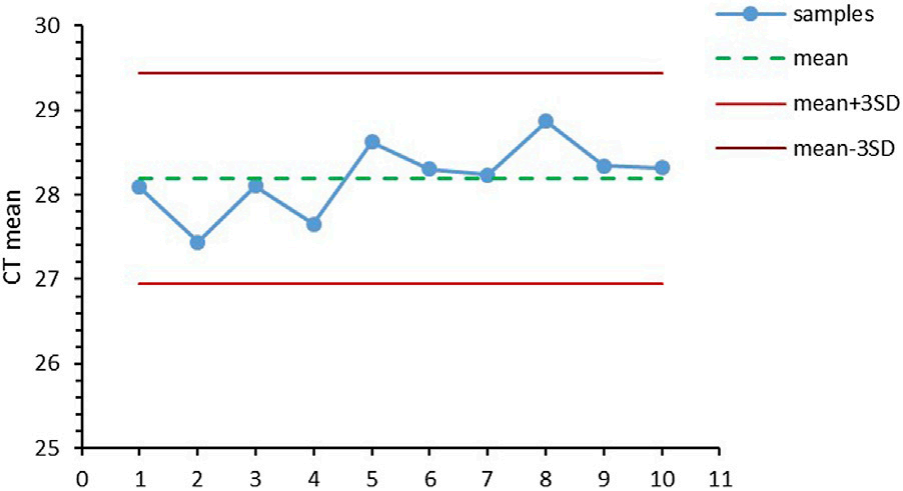
Repeated experimental results of automated EV miRNA isolation and detection (*n* = 10).

Different matrices were compared to study the influence factors for EV miRNA extraction, including PBS, DMEM, and exosome-free FBS. As shown in Figure 4E, the CT values for miRNA-21 extracted from the three matrices were the same, without significant difference. The results showed that the method for EV miRNA isolation by magnetic beads has wide adaptability. To compare the recovery rate for the automatic instrument and manual method, we calculated the weight ratio of the weight of the beads before and after the experiment. As shown in Figure 4F, the recovery rate for the magnetic beads on the automatic instrument reached up to 96%, while the highest recovery rate for the magnetic beads by the manual method was only 86%. The SD value for the instrumental method was also lower than that of the manual method, indicating that the recovery and repeatability of the instrumental method was better than that of the manual method. The repeatability of methods is particularly important in clinical testing. We used several human plasma samples (*n* = 10) to perform EV miRNA extraction and RT-qPCR detection of miRNA-21. All CT values were between mean ± 3 SD (Figure 5). The extraction of EV miRNA was confirmed to have good reproducibility by the repeated test.

### Extracellular vesicle miRNA-21 analysis

**FIGURE 6:**
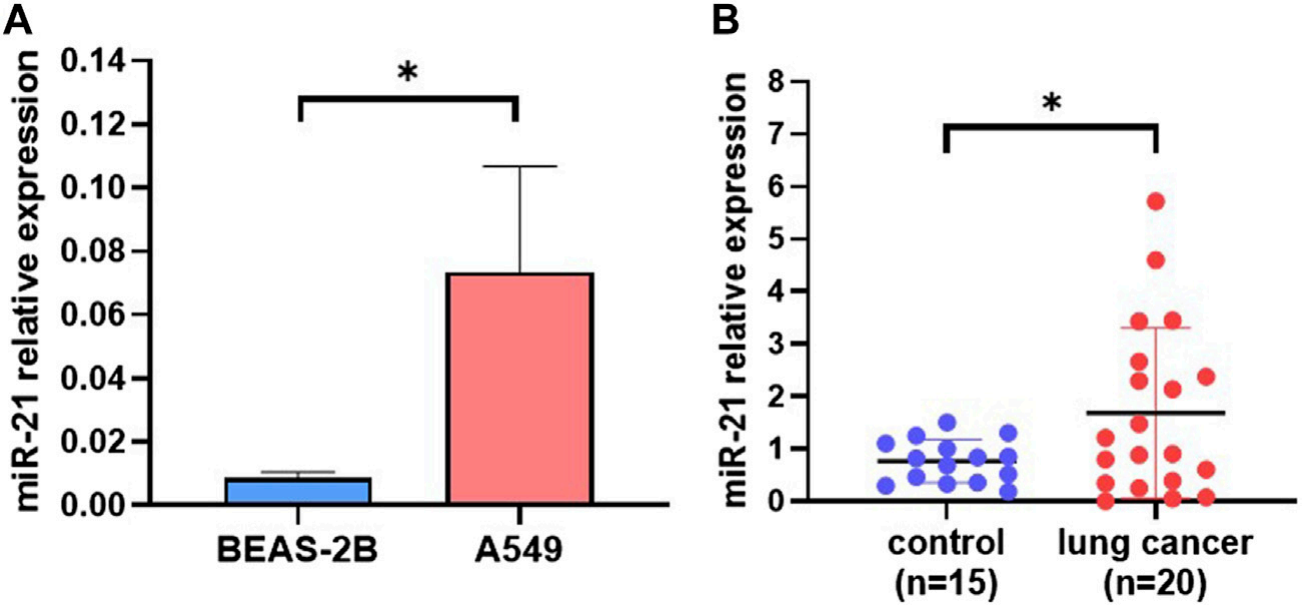
EV miRNA-21 analysis from different samples. **(A)** Difference in EV-derived miRNA-21 expression between A549 cells and Beas-2B cells (*p < 0.05). **(B)** Difference in plasma EV–derived miRNA-21 expression between healthy persons (*n* = 15) and lung cancer patients (*n* = 20) (*p < 0.05).

To demonstrate the possibility of applying this extraction method for research and clinical application, first, we verified it in a cell culture medium of human normal lung cell BEAS-2B and lung cancer cell A549. We found that the expression level of miRNA-21 in the EVs derived from A549 cells was significantly higher than that for the BEAS-2B cells (Figure 6A). Moreover, we compared the EV miRNA-21 expression from plasma after EV miRNA was extracted by the automated method based on Fe_3_O_4_@TiO_2_. As shown in Figure 6B, the expression level of EV-derived miRNA-21 in the plasma from lung cancer patients was significantly higher than that of healthy persons. These results indicate that miRNA-21 can be used as a marker to distinguish lung cancer, which is consistent with the results from previous studies (Jin et al., 2017). This method can be used to detect clinical samples with advantages of more speed and convenience.

## Discussion

The challenges of EV isolation methods, such as complicated operation and low recovery rate, limit research and application of EV miRNA. Recently, TiO_2_ was discovered to have the ability to capture EVs. The Ti^4+^ on the surface of Fe_3_O_4_@TiO_2_ can combine with the phosphate groups on the EV membrane (Pang et al., 2020). The recovery rate for the EVs was up to 80% based on Fe_3_O_4_@TiO_2_ in 10 min. By contrast, the EV recovery rate for the ultracentrifugation method was less than 20% (Pang et al., 2020). Our research results showed that Fe_3_O_4_@TiO_2_ successfully enriched EVs. The combination of magnetic beads and the automation method can obtain higher efficiency within less time (Supplementary Table S2) (Li et al., 2013).

The traditional TRIzol method or commercial kit is usually used to extract EV miRNA, which requires a large sample, cumbersome operation, and has poor repeatability. Considering that the enrichment of Fe_3_O_4_@TiO_2_ reduced the components of proteins and nucleic acid pollutions, direct heat-lysis on the beads to extract RNA minimized the miRNA loss in a short time. The heat-lysis method has a significantly larger amount of EV miRNA. It has been reported that the RT-qPCR samples can be prepared by heat treatment of swab samples to diagnose COVID-19 (Bruce et al., 2020; Fomsgaard and Rosenstierne, 2020). Our experimental results show that miRNA-21 is highly expressed in EVs of lung cancer cells and plasma of lung cancer patients, consistent with other research studies (Bica-Pop et al., 2018).

In conclusion, we established a rapid and automatic EV miRNA extraction technology based on Fe_3_O_4_@TiO_2_ beads, which will provide a very efficient tool for the clinical detection of EV miRNA. This method not only reduced the loss of nucleic acid during extraction steps but also avoided manual errors through automated procedures. We can effectively extract miRNA from only 50 μl plasma samples for detection, which is superior to TRIzol extraction technology and commercial kit. At present, the EV nucleic acid extraction method is relatively complex, and there is a large loss of nucleic acid in the operation process, which usually requires a 200 μl to 1 ml sample. The automated extraction method in our research has the advantages of high throughput, is fast, and requires less number of samples, which is very suitable for point-of-care testing (POCT) in clinical use. In addition to nucleic acid extraction, this method can be easily extended and used for the detection of various types of markers such as protein and lipid.

## Data Availability

The original contributions presented in the study are included in the article/Supplementary Material; further inquiries can be directed to the corresponding author.
