# Supplementary material for: Highly efficient and automated isolation technology for extracellular vesicles microRNA
Source: Front Bioeng Biotechnol. 2022 Aug 10;10:948757. doi: 10.3389/fbioe.2022.948757 (PMC9399425; doi:10.3389/fbioe.2022.948757)
Supplement: Supplementary file 1 [file Table1.DOCX]

Supplementary Material

## Supplementary Figures


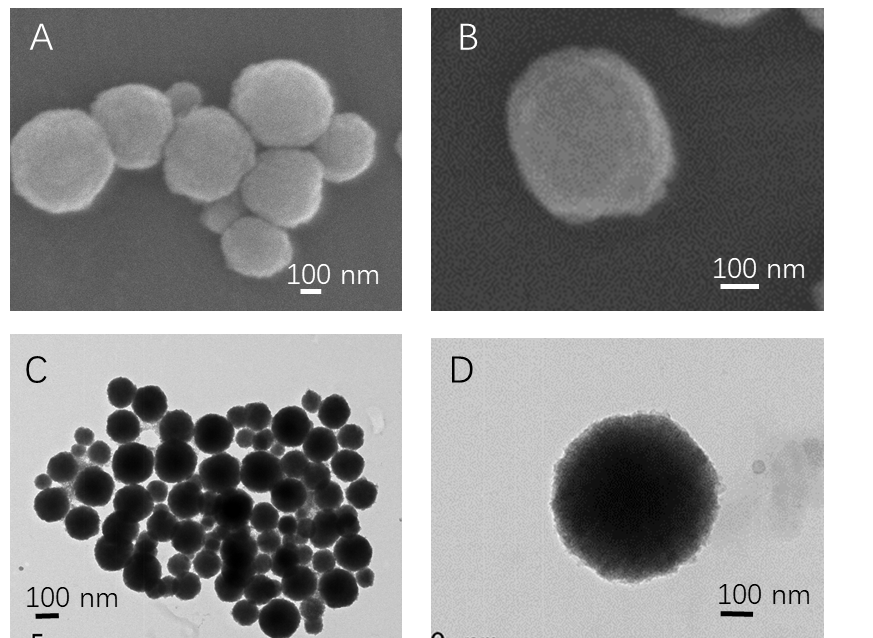


**Supplementary Figure 1.** Characterization of Fe_3_O_4_@TiO_2_. (**A)** SEM images of multiple Fe_3_O_4_@TiO_2_. (**B)** SEM images of single Fe_3_O_4_@TiO_2_. (**C)** TEM images of multiple Fe_3_O_4_@TiO_2_. (**D)** TEM images of single Fe_3_O_4_@TiO_2_.


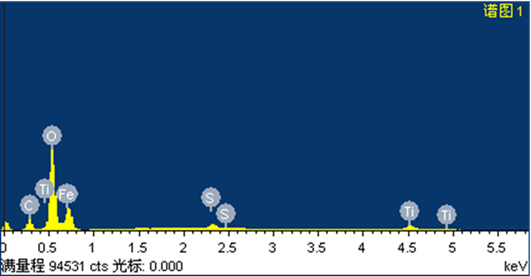


**Supplementary Figure 2.** The energy dispersive X-ray (EDX) spectrum of Fe_3_O_4_@TiO_2_.


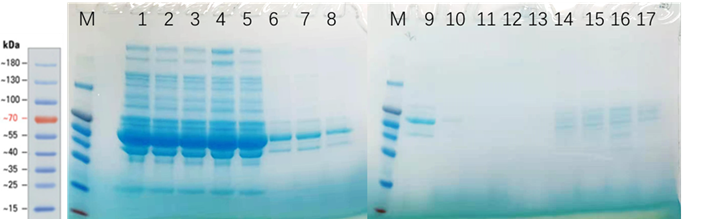


**Supplementary Figure 3.** SDS-PAGE result of EVs isolation by Fe_3_O_4_@TiO_2_. **M** is protein marker; **1** is cell culture medium of A549 cells; **2-5** is supernatant separated by magnetic separation after capture; **6-9** is supernatant separated by magnetic separation after the first cleaning; **10-13** is supernatant separated by magnetic separation after the third cleaning; **14-17** is the lysate from Fe_3_O_4_@TiO_2_ beads. The gel was stained with coomassie bright blue.


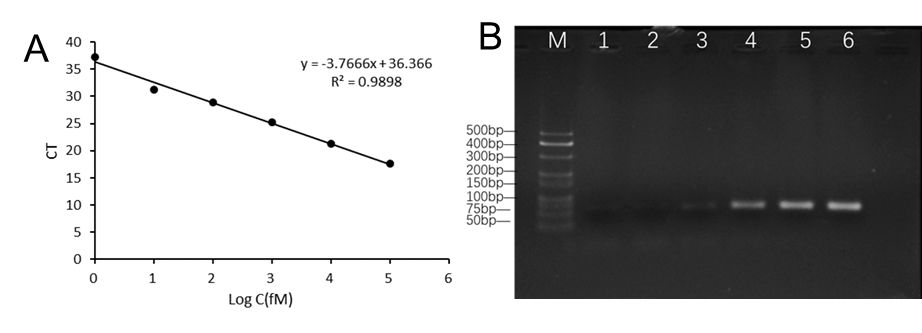


**Supplementary Figure 4. (A)** RT-qPCR standard curve of miRNA-21. **(B)** PCR products electrophoresis result. **M**: DNA marker; **1-6**: PCR products including 1 fM, 10 fM, 100 fM, 1 pM, 10 pM and 100 pM of miRNA-21 template.

**Supplementary Table 1.** The sequence of RT-qPCR primers for miRNA-21 detection.

| **primer** | **sequence** | |
| --- | --- | --- |
| Stem-loop RT | | 5'-GTCGTATCCAGTGCAGGGTCCGAGGTATTCGCACTGGATACG-3' |
| Forward primer | | 5'-GCGCGTAGCTTATCAGACTGA-3' |
| Reverse primer | | 5'-AGTGCAGGGTCCGAGGTATT-3' |

**Supplementary Table 2.** Comparison of EVs miRNA extraction methods.

| **Method** | **EVs enrichment time** | **miRNA extraction time** | **Manual operation time** |
| --- | --- | --- | --- |
| Automatic Isolation of EVs miRNA Based on Fe_3_O_4_@TiO_2_ (this article) | 15 min | 5 min | _ |
| Ultracentrifugation | 3 h | — | ~1 h |
| Precipitation method | >1 h | — | ~30 min |
| Trizol | — | 3 h | ~1 h |
| miRNeasy Serum/Plasma Advanced Kit | — | 1 h | ~30 min |
